# 2-Amino-5-methyl­pyridinium 3-hy­droxy­pyridine-2-carboxyl­ate

**DOI:** 10.1107/S1600536813016322

**Published:** 2013-06-19

**Authors:** Abbas Farhadikoutenaei, Kaliyaperumal Thanigaimani, Suhana Arshad, Ibrahim Abdul Razak

**Affiliations:** a School of Physics, Universiti Sains Malaysia, 11800 USM, Penang, Malaysia; b Department of Physics, Faculty of Science, University of Mazandaran, Babolsar, Iran

## Abstract

In the 3-hy­droxy­picolinate anion of the title salt, C_6_H_9_N_2_^+^·C_6_H_4_NO_3_^−^, an intra­molecular O—H⋯O hydrogen bond with an *S*(6) graph-set motif is formed, so that the anion is essentially planar, with a dihedral angle of 9.55 (9)° between the pyridine ring and the carboxyl­ate group. In the crystal, the cations and anions are linked *via* N—H⋯O hydrogen bonds, forming a centrosymmetric 2 + 2 aggregate with *R*_2_^2^(8) and *R*_4_^2^(8) ring motifs. The crystal structure also features N—H⋯N and weak C—H⋯π inter­actions.

## Related literature

For details of non-covalent inter­actions, see: Desiraju (2007 ▶); Aakeroy & Seddon (1993 ▶). For related structures, see: Nahringbauer & Kvick (1977 ▶); Robert *et al.* (2001 ▶); Thanigaimani *et al.* (2010 ▶, 2013 ▶). For hydrogen-bond motifs, see: Bernstein *et al.* (1995 ▶). For bond-length data, see: Allen *et al.* (1987 ▶). For stability of the temperature controller used for the data collection, see: Cosier & Glazer (1986 ▶).
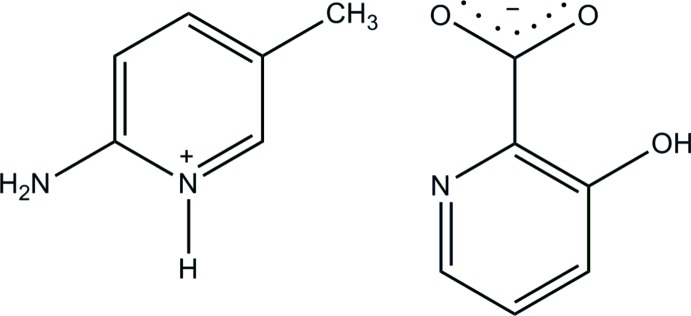


## Experimental

### 

#### Crystal data

C_6_H_9_N_2_^+^·C_6_H_4_NO_3_^−^
*M* *_r_* = 247.25
Monoclinic, 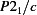

*a* = 7.3443 (4) Å
*b* = 16.4321 (9) Å
*c* = 10.8235 (5) Å
β = 118.250 (3)°
*V* = 1150.62 (10) Å^3^
*Z* = 4
Mo *K*α radiation
μ = 0.11 mm^−1^
*T* = 100 K
0.58 × 0.29 × 0.16 mm

#### Data collection

Bruker SMART APEXII DUO CCD area-detector diffractometer
Absorption correction: multi-scan (*SADABS*; Bruker, 2009 ▶) *T*_min_ = 0.942, *T*_max_ = 0.984
15996 measured reflections
4132 independent reflections
3596 reflections with *I* > 2σ(*I*)
*R*_int_ = 0.020

#### Refinement

*R*[*F* ^2^ > 2σ(*F* ^2^)] = 0.036
*wR*(*F* ^2^) = 0.115
*S* = 1.04
4132 reflections
180 parameters
H atoms treated by a mixture of independent and constrained refinement
Δρ_max_ = 0.47 e Å^−3^
Δρ_min_ = −0.22 e Å^−3^

### 

Data collection: *APEX2* (Bruker, 2009 ▶); cell refinement: *SAINT* (Bruker, 2009 ▶); data reduction: *SAINT*; program(s) used to solve structure: *SHELXTL* (Sheldrick, 2008 ▶); program(s) used to refine structure: *SHELXTL*; molecular graphics: *SHELXTL*; software used to prepare material for publication: *SHELXTL* and *PLATON* (Spek, 2009 ▶).

## Supplementary Material

Crystal structure: contains datablock(s) global, I. DOI: 10.1107/S1600536813016322/is5281sup1.cif

Structure factors: contains datablock(s) I. DOI: 10.1107/S1600536813016322/is5281Isup2.hkl

Supplementary material file. DOI: 10.1107/S1600536813016322/is5281Isup3.cml

Additional supplementary materials:  crystallographic information; 3D view; checkCIF report

## Figures and Tables

**Table 1:** Hydrogen-bond geometry (Å, °) *Cg*1 is the centroid of the N1/C1–C5 ring.

*D*—H⋯*A*	*D*—H	H⋯*A*	*D*⋯*A*	*D*—H⋯*A*
O1—H1*O*1⋯O2	0.93 (2)	1.66 (2)	2.5239 (10)	152 (2)
N3—H2*N*3⋯O3^i^	0.885 (15)	1.969 (15)	2.8504 (11)	174.0 (14)
N3—H1*N*3⋯O3^ii^	0.859 (14)	2.248 (15)	2.8093 (10)	123.0 (12)
N3—H1*N*3⋯N1^ii^	0.859 (14)	2.416 (14)	3.2481 (10)	163.2 (13)
N2—H1*N*2⋯O2^i^	0.943 (16)	1.796 (16)	2.7327 (10)	171.4 (13)
C9—H9*A*⋯*Cg*1	0.95	2.59	3.4702 (10)	154
C11—H11*A*⋯*Cg*1^iii^	0.95	2.71	3.3956 (8)	130

Symmetry codes: (i) 
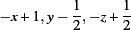
; (ii) 
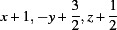
; (iii) 
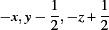
.

**Fig. 1.**
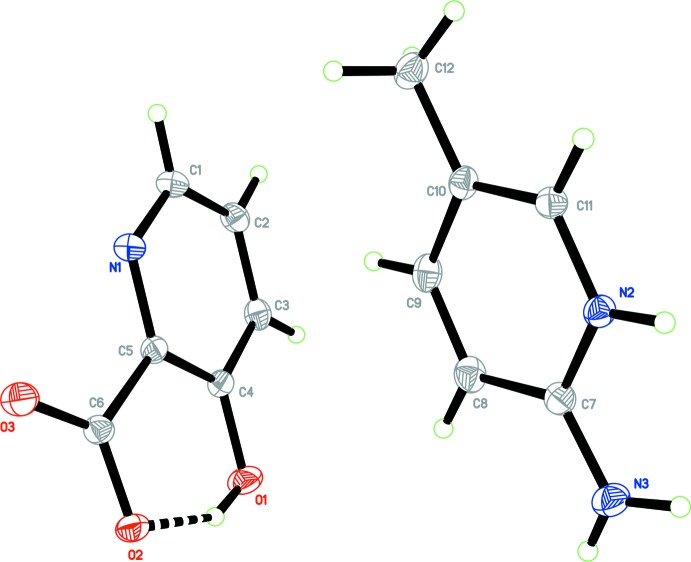
The molecular structure of the title compound with atom labels with 50% probability displacement ellipsoids.

**Fig. 2.**
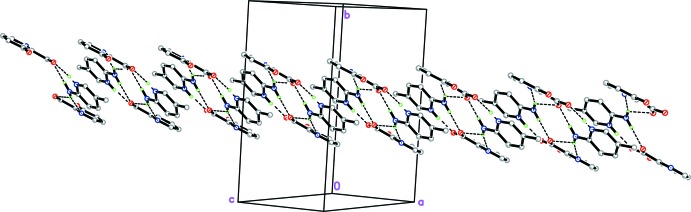
The crystal packing of the title compound. The H atoms not involved in the intermolecular interactions (dashed lines) have been omitted for clarity.

## supplementary crystallographic information

### Comment

Supramolecular architectures assembled *via* various delicate noncovalent interactions such as hydrogen bonds, π–π stacking and electrostatic interactions, *etc*., have attracted intense interest in recent years because of their fascinating structural diversity and potential applications for functional materials (Desiraju, 2007). Especially, the application of intermolecular hydrogen bonds is a well known and efficient tool in the field of organic crystal design owing to its strength and directional properties (Aakeroy & Seddon, 1993). In order to study potential hydrogen bonding interactions, the crystal structure determination of the title compound (I) was carried out.

The asymmetric unit (Fig. 1) contains one 2-amino-5-methylpyridinium cation and one 3-hydroxypicolinate anion. An intramolecular O1—H1O1···O2 hydrogen bond in the 3-hydroxypicolinate anion generates an S(6) ring motif. (Bernstein *et al.*, 1995). This motif is also observed in the crystal structure of acetoguanaminium 3-hydroxypicolinate monohydrate (Thanigaimani *et al.*, 2010). The proton transfers from the one of the carboxyl group oxygen atom (O2) to atom N1 of 2-amino-5-methylpyrimidine resulted in the widening of C7—N2—C11 angle of the pyridinium ring to 122.89 (7)°, compared to the corresponding angle of 117.4 (3)° in neutral 2-amino-5-methylpyridine (Nahringbauer & Kvick, 1977). The 2-amino-5-methylpyridinium cation is essentially planar, with a maximum deviation of 0.011 (1) Å for atom C9. The bond lengths (Allen *et al.*, 1987) and angles are normal.

In the crystal packing (Fig. 2), the protonated N2 atom and a nitrogen atom of the 2-amino group (N3) are hydrogen-bonded to the carboxylate oxygen atoms (O2 and O3) *via* a pair of intramolecular N2—H1N2···O2^i^ and N3—H2N3···O3^i^ hydrogen bonds (symmetry code in Table 1), forming a ring motif *R*_2_^2^(8) (Bernstein *et al.*, 1995). These motifs are linked by N3—H1N3···O3^ii^ hydrogen bonds (symmetry code in Table 1), forming a ring spanning the centre of symmetry at (1, -3/2, 1/2) to produce a DDAA array (where D is a hydrogen-bond donor and A is a hydrogen-bond acceptor) of four hydrogen bonds. This set of fused rings can be represented by the graph-set notations *R*_2_^2^(8), *R*_4_^2^(8) and *R*_2_^2^(8) arrangement. This type of motif has been reported in the crystal structures of trimethoprim hydrogen glutarate (Robert *et al.*, 2001), acetoguanaminium 3-hydroxypicolinate monohydrate (Thanigaimani *et al.*, 2010) and 2-amino-6-methylpyridinium 3-chlorobenzoate (Thanigaimani *et al.*, 2013). The 2-aminogroup at N3 forms a bifurcated hydrogen bond (Table 1) with carboxyl atom O3^ii^ and atom N1^ii^ of a 3-hydroxypicolinate anion [graph-set *R*_1_^2^(5)]. The crystal structure is further stabilized by weak C—H···π interactions (Table 1) involving the N1/C1–C5 (centroid *Cg*1) ring.

### Experimental

Hot methanol solutions (20 ml) of 2-amino5-methylpyridine (54 mg, Aldrich) and 3-hydoxypicolinic acid (34 mg, Aldrich) were mixed and warmed over a heating magnetic stirrer hotplate for a few minutes. The resulting solution was allowed to cool slowly at room temperature and crystals of the title compound (I) appeared after a few days.

### Refinement

O- and N-bound H atoms were located in a difference Fourier map and were refined freely [O—H = 0.926 (19) Å and N—H = 0.859 (14)–0.927 (15) Å]. The remaining hydrogen atoms were positioned geometrically (C—H = 0.95–0.98 Å) and were refined using a riding model, with *U*_iso_(H) = 1.2*U*_eq_(C) or 1.5*U*_eq_(methyl C). A rotating group model was used for the methyl group. Five outliers were omitted (2 4 1, 2 1 5, 1 0 2, 3 3 4 and 1 6 0) in the final refinement.

### Crystal data

**Table:**

C_6_H_9_N_2_^+^·C_6_H_4_NO_3_^−^	*F*(000) = 520
*M**_r_* = 247.25	*D*_x_ = 1.427 Mg m^−^^3^
Monoclinic, *P*2_1_/*c*	Mo *K*α radiation, λ = 0.71073 Å
Hall symbol: -P 2ybc	Cell parameters from 8234 reflections
*a* = 7.3443 (4) Å	θ = 2.5–32.6°
*b* = 16.4321 (9) Å	µ = 0.11 mm^−^^1^
*c* = 10.8235 (5) Å	*T* = 100 K
β = 118.250 (3)°	Block, colourless
*V* = 1150.62 (10) Å^3^	0.58 × 0.29 × 0.16 mm
*Z* = 4	

### Data collection

**Table:**

Bruker SMART APEXII DUO CCD area-detector diffractometer	4132 independent reflections
Radiation source: fine-focus sealed tube	3596 reflections with *I* > 2σ(*I*)
Graphite monochromator	*R*_int_ = 0.020
φ and ω scans	θ_max_ = 32.6°, θ_min_ = 2.5°
Absorption correction: multi-scan (*SADABS*; Bruker, 2009)	*h* = −11→9
*T*_min_ = 0.942, *T*_max_ = 0.984	*k* = −24→24
15996 measured reflections	*l* = −16→16

### Refinement

**Table:**

Refinement on *F*^2^	Primary atom site location: structure-invariant direct methods
Least-squares matrix: full	Secondary atom site location: difference Fourier map
*R*[*F*^2^ > 2σ(*F*^2^)] = 0.036	Hydrogen site location: inferred from neighbouring sites
*wR*(*F*^2^) = 0.115	H atoms treated by a mixture of independent and constrained refinement
*S* = 1.04	*w* = 1/[σ^2^(*F*_o_^2^) + (0.0686*P*)^2^ + 0.2463*P*] where *P* = (*F*_o_^2^ + 2*F*_c_^2^)/3
4132 reflections	(Δ/σ)_max_ = 0.001
180 parameters	Δρ_max_ = 0.47 e Å^−^^3^
0 restraints	Δρ_min_ = −0.22 e Å^−^^3^

### Special details

**Table:**

Experimental. The crystal was placed in the cold stream of an Oxford Cryosystems Cobra open-flow nitrogen cryostat (Cosier & Glazer, 1986) operating at 100.0 (1) K.
Geometry. All e.s.d.'s (except the e.s.d. in the dihedral angle between two l.s. planes) are estimated using the full covariance matrix. The cell e.s.d.'s are taken into account individually in the estimation of e.s.d.'s in distances, angles and torsion angles; correlations between e.s.d.'s in cell parameters are only used when they are defined by crystal symmetry. An approximate (isotropic) treatment of cell e.s.d.'s is used for estimating e.s.d.'s involving l.s. planes.
Refinement. Refinement of *F*^2^ against ALL reflections. The weighted *R*-factor *wR* and goodness of fit *S* are based on *F*^2^, conventional *R*-factors *R* are based on *F*, with *F* set to zero for negative *F*^2^. The threshold expression of *F*^2^ > σ(*F*^2^) is used only for calculating *R*-factors(gt) *etc*. and is not relevant to the choice of reflections for refinement. *R*-factors based on *F*^2^ are statistically about twice as large as those based on *F*, and *R*- factors based on ALL data will be even larger.

### Fractional atomic coordinates and isotropic or equivalent isotropic displacement parameters (Å^2^)

**Table:**

	*x*	*y*	*z*	*U*_iso_*/*U*_eq_	
O1	0.38403 (10)	0.83927 (4)	0.52572 (6)	0.02228 (14)	
O2	0.41088 (9)	0.89008 (4)	0.31488 (7)	0.01954 (13)	
O3	0.12614 (10)	0.90599 (4)	0.10825 (6)	0.01974 (13)	
N1	−0.10602 (10)	0.82189 (4)	0.20289 (7)	0.01502 (13)	
C1	−0.22027 (12)	0.78730 (5)	0.25475 (8)	0.01684 (14)	
H1A	−0.3598	0.7738	0.1920	0.020*	
C2	−0.14303 (13)	0.77021 (4)	0.39745 (8)	0.01669 (14)	
H2A	−0.2297	0.7465	0.4307	0.020*	
C3	0.06046 (13)	0.78818 (4)	0.48931 (8)	0.01635 (14)	
H3A	0.1159	0.7773	0.5867	0.020*	
C4	0.18388 (12)	0.82274 (4)	0.43669 (8)	0.01437 (14)	
C5	0.09352 (11)	0.83951 (4)	0.29191 (7)	0.01291 (13)	
C6	0.21674 (12)	0.88142 (4)	0.23125 (8)	0.01460 (14)	
N2	0.39598 (10)	0.50115 (4)	0.27137 (7)	0.01444 (13)	
N3	0.70889 (12)	0.55236 (5)	0.44004 (8)	0.02005 (14)	
C7	0.50370 (12)	0.55708 (4)	0.37131 (8)	0.01518 (14)	
C8	0.38865 (14)	0.61797 (5)	0.39723 (8)	0.01833 (15)	
H8A	0.4581	0.6590	0.4655	0.022*	
C9	0.17762 (14)	0.61731 (5)	0.32352 (9)	0.01811 (15)	
H9A	0.1020	0.6576	0.3431	0.022*	
C10	0.06826 (12)	0.55828 (4)	0.21858 (8)	0.01489 (14)	
C11	0.18508 (12)	0.50126 (4)	0.19649 (8)	0.01427 (14)	
H11A	0.1179	0.4606	0.1272	0.017*	
C12	−0.16380 (13)	0.56034 (5)	0.13631 (9)	0.01984 (16)	
H12A	−0.2134	0.5109	0.0793	0.030*	
H12B	−0.2072	0.6082	0.0750	0.030*	
H12C	−0.2220	0.5633	0.2011	0.030*	
H2N3	0.766 (2)	0.5071 (9)	0.4311 (15)	0.030 (3)*	
H1N3	0.779 (2)	0.5866 (8)	0.5053 (15)	0.029 (3)*	
H1O1	0.437 (3)	0.8585 (12)	0.469 (2)	0.058 (5)*	
H1N2	0.465 (2)	0.4611 (9)	0.2494 (15)	0.036 (4)*	

### Atomic displacement parameters (Å^2^)

**Table:**

	*U*^11^	*U*^22^	*U*^33^	*U*^12^	*U*^13^	*U*^23^
O1	0.0138 (3)	0.0296 (3)	0.0167 (3)	−0.0022 (2)	0.0017 (2)	0.0055 (2)
O2	0.0115 (3)	0.0238 (3)	0.0219 (3)	−0.0005 (2)	0.0067 (2)	0.0054 (2)
O3	0.0173 (3)	0.0264 (3)	0.0151 (3)	−0.0030 (2)	0.0073 (2)	0.0024 (2)
N1	0.0137 (3)	0.0162 (3)	0.0150 (3)	−0.0018 (2)	0.0067 (2)	−0.0012 (2)
C1	0.0144 (3)	0.0181 (3)	0.0183 (3)	−0.0032 (2)	0.0080 (3)	−0.0014 (2)
C2	0.0187 (3)	0.0156 (3)	0.0190 (3)	−0.0011 (3)	0.0116 (3)	0.0000 (2)
C3	0.0202 (4)	0.0150 (3)	0.0148 (3)	0.0006 (2)	0.0090 (3)	0.0010 (2)
C4	0.0136 (3)	0.0139 (3)	0.0139 (3)	0.0007 (2)	0.0050 (3)	0.0007 (2)
C5	0.0123 (3)	0.0130 (3)	0.0136 (3)	0.0005 (2)	0.0063 (3)	0.0003 (2)
C6	0.0135 (3)	0.0148 (3)	0.0165 (3)	−0.0001 (2)	0.0079 (3)	−0.0001 (2)
N2	0.0137 (3)	0.0146 (3)	0.0148 (3)	0.0010 (2)	0.0066 (2)	−0.0013 (2)
N3	0.0148 (3)	0.0217 (3)	0.0187 (3)	−0.0003 (2)	0.0039 (3)	−0.0020 (2)
C7	0.0162 (3)	0.0151 (3)	0.0130 (3)	−0.0005 (2)	0.0058 (3)	0.0006 (2)
C8	0.0217 (4)	0.0153 (3)	0.0162 (3)	0.0011 (3)	0.0076 (3)	−0.0024 (2)
C9	0.0216 (4)	0.0155 (3)	0.0185 (3)	0.0044 (3)	0.0105 (3)	0.0004 (2)
C10	0.0154 (3)	0.0150 (3)	0.0153 (3)	0.0023 (2)	0.0082 (3)	0.0028 (2)
C11	0.0141 (3)	0.0147 (3)	0.0141 (3)	−0.0003 (2)	0.0067 (3)	−0.0001 (2)
C12	0.0152 (3)	0.0223 (3)	0.0230 (4)	0.0034 (3)	0.0098 (3)	0.0051 (3)

### Geometric parameters (Å, º)

**Table:**

O1—C4	1.3499 (9)	N2—H1N2	0.927 (15)
O1—H1O1	0.926 (19)	N3—C7	1.3301 (10)
O2—C6	1.2848 (9)	N3—H2N3	0.883 (15)
O3—C6	1.2408 (9)	N3—H1N3	0.859 (14)
N1—C1	1.3372 (10)	C7—C8	1.4207 (11)
N1—C5	1.3502 (10)	C8—C9	1.3668 (12)
C1—C2	1.3989 (11)	C8—H8A	0.9500
C1—H1A	0.9500	C9—C10	1.4187 (11)
C2—C3	1.3793 (11)	C9—H9A	0.9500
C2—H2A	0.9500	C10—C11	1.3657 (10)
C3—C4	1.3990 (11)	C10—C12	1.5040 (11)
C3—H3A	0.9500	C11—H11A	0.9500
C4—C5	1.4098 (10)	C12—H12A	0.9800
C5—C6	1.5122 (10)	C12—H12B	0.9800
N2—C7	1.3529 (10)	C12—H12C	0.9800
N2—C11	1.3666 (10)		
			
C4—O1—H1O1	104.5 (12)	C7—N3—H1N3	120.3 (10)
C1—N1—C5	118.45 (6)	H2N3—N3—H1N3	120.5 (13)
N1—C1—C2	122.80 (7)	N3—C7—N2	119.11 (7)
N1—C1—H1A	118.6	N3—C7—C8	123.57 (7)
C2—C1—H1A	118.6	N2—C7—C8	117.32 (7)
C3—C2—C1	119.19 (7)	C9—C8—C7	119.69 (7)
C3—C2—H2A	120.4	C9—C8—H8A	120.2
C1—C2—H2A	120.4	C7—C8—H8A	120.2
C2—C3—C4	118.90 (7)	C8—C9—C10	121.91 (7)
C2—C3—H3A	120.6	C8—C9—H9A	119.0
C4—C3—H3A	120.6	C10—C9—H9A	119.0
O1—C4—C3	119.18 (7)	C11—C10—C9	116.40 (7)
O1—C4—C5	122.33 (7)	C11—C10—C12	122.79 (7)
C3—C4—C5	118.49 (7)	C9—C10—C12	120.80 (7)
N1—C5—C4	122.15 (7)	C10—C11—N2	121.78 (7)
N1—C5—C6	117.28 (6)	C10—C11—H11A	119.1
C4—C5—C6	120.53 (7)	N2—C11—H11A	119.1
O3—C6—O2	124.93 (7)	C10—C12—H12A	109.5
O3—C6—C5	119.13 (7)	C10—C12—H12B	109.5
O2—C6—C5	115.92 (6)	H12A—C12—H12B	109.5
C7—N2—C11	122.89 (6)	C10—C12—H12C	109.5
C7—N2—H1N2	120.2 (9)	H12A—C12—H12C	109.5
C11—N2—H1N2	116.9 (9)	H12B—C12—H12C	109.5
C7—N3—H2N3	117.5 (9)		
			
C5—N1—C1—C2	1.36 (11)	N1—C5—C6—O2	−173.26 (6)
N1—C1—C2—C3	−1.19 (12)	C4—C5—C6—O2	9.01 (10)
C1—C2—C3—C4	−0.26 (11)	C11—N2—C7—N3	179.59 (7)
C2—C3—C4—O1	−178.95 (7)	C11—N2—C7—C8	−0.11 (11)
C2—C3—C4—C5	1.41 (11)	N3—C7—C8—C9	−178.71 (7)
C1—N1—C5—C4	−0.11 (11)	N2—C7—C8—C9	0.97 (11)
C1—N1—C5—C6	−177.80 (6)	C7—C8—C9—C10	−1.42 (12)
O1—C4—C5—N1	179.10 (7)	C8—C9—C10—C11	0.95 (11)
C3—C4—C5—N1	−1.28 (11)	C8—C9—C10—C12	−178.32 (7)
O1—C4—C5—C6	−3.29 (11)	C9—C10—C11—N2	−0.06 (11)
C3—C4—C5—C6	176.34 (6)	C12—C10—C11—N2	179.19 (6)
N1—C5—C6—O3	8.08 (10)	C7—N2—C11—C10	−0.35 (11)
C4—C5—C6—O3	−169.65 (7)		

### Hydrogen-bond geometry (Å, º)

*Cg*1 is the centroid of the N1/C1–C5 ring.

**Table:**

*D*—H···*A*	*D*—H	H···*A*	*D*···*A*	*D*—H···*A*
O1—H1*O*1···O2	0.93 (2)	1.66 (2)	2.5239 (10)	152 (2)
N3—H2*N*3···O3^i^	0.885 (15)	1.969 (15)	2.8504 (11)	174.0 (14)
N3—H1*N*3···O3^ii^	0.859 (14)	2.248 (15)	2.8093 (10)	123.0 (12)
N3—H1*N*3···N1^ii^	0.859 (14)	2.416 (14)	3.2481 (10)	163.2 (13)
N2—H1*N*2···O2^i^	0.943 (16)	1.796 (16)	2.7327 (10)	171.4 (13)
C9—H9*A*···*Cg*1	0.95	2.59	3.4702 (10)	154
C11—H11*A*···*Cg*1^iii^	0.95	2.71	3.3956 (8)	130

Symmetry codes: (i) −*x*+1, *y*−1/2, −*z*+1/2; (ii) *x*+1, −*y*+3/2, *z*+1/2; (iii) −*x*, *y*−1/2, −*z*+1/2.
